# Pringle Maneuver in Extended Liver Resection: A propensity score analysis

**DOI:** 10.1038/s41598-020-64596-y

**Published:** 2020-06-01

**Authors:** Mohammed Al-Saeedi, Omid Ghamarnejad, Elias Khajeh, Saeed Shafiei, Roozbeh Salehpour, Mohammad Golriz, Markus Mieth, Karl Heinz Weiss, Thomas Longerich, Katrin Hoffmann, Markus W. Büchler, Arianeb Mehrabi

**Affiliations:** 1 Department of General, Visceral, and Transplantation Surgery, University of Heidelberg, Heidelberg, Germany; 2 Liver Cancer Center Heidelberg (LCCH), Heidelberg, Germany; 3 Department of Gastroenterology and Hepatology, University of Heidelberg, Heidelberg, Germany; 4 Institute of Pathology, University of Heidelberg, Heidelberg, Germany

**Keywords:** Liver, Medical research

## Abstract

Despite the ongoing decades-long controversy, Pringle maneuver (PM) is still frequently used by hepatobiliary surgeons during hepatectomy. The aim of this study was to investigate the effect of PM on intraoperative blood loss, morbidity, and posthepatectomy hemorrhage (PHH). A series of 209 consecutive patients underwent extended hepatectomy (EH) (≥5 segment resection). The association of PM with perioperative outcomes was evaluated using multivariate analysis with a propensity score method to control for confounding. Fifty patients underwent PM with a median duration of 19 minutes. Multivariate analysis revealed that risk of excessive intraoperative bleeding (≥1500 ml; odds ratio [OR] 0.27, 95%-confidence interval [CI] 0.10–0.70, p = 0.007), major morbidity (OR 0.41, 95%-CI 0.18–0.97, p = 0.041), and PHH (OR 0.22, 95%-CI 0.06–0.79, p = 0.021) were significantly lower in PM group after EH. Furthermore, there was no significant difference in 3-year recurrence-free-survival between groups. PM is associated with lower intraoperative bleeding, PHH, and major morbidity risk after EH. Performing PM does not increase posthepatectomy liver failure and does not affect recurrence rate. Therefore, PM seems to be justified in EH.

## Introduction

Extended hepatectomy (EH) is the only curative treatment option for patients with large primary or bilobar metastatic liver malignancies^1^. Better patient selection and developments in surgical techniques and instruments have increased the number and safety of EH^2,3^. However, the risk of complications such as intraoperative bleeding, especially in patients with large tumors or tumors near to major vessels, is still high. These factors are associated with poorer postoperative outcomes^4,5^. Patients with massive intraoperative blood loss have a higher rate of posthepatectomy morbidity and mortality^2^ and lower recurrence-free survival due to blood transfusion^6^. Therefore, reducing intraoperative bleeding during EH and reducing the amount of blood products transfused are important points in liver surgery.

Despite the ongoing controversy regarding the advantages and disadvantages of hepatic inflow control during hepatectomy, the Pringle maneuver (PM) remains the most commonly used and evidence-based method of hepatic inflow control^7^. The PM significantly decreases intraoperative blood loss, the amount of blood products transfused, and operation time, especially when performed in combination with low central venous pressure^8–10^. Nevertheless, there is no evidence that the PM can reduce posthepatectomy morbidity and mortality^11,12^; in fact, the PM may result in ischemia-reperfusion injury of the liver, which negatively affects hepatocyte metabolism, thereby increasing the rate of posthepatectomy liver failure (PHLF)^13,14^.

Despite several studies investigating the role of the PM in liver resection, the effects of the PM on intra- and postoperative outcomes have not been investigated exclusively in EH, which has a higher risk of intraoperative bleeding than minor hepatectomies^15^. In addition, application of the PM in minimal invasive surgery has recently increased, along with laparoscopic and robotic major hepatectomies, to prevent uncontrolled bleeding and conversion to open surgery^16–18^. Therefore, the role of the PM in EH with higher risks of intraoperative, postoperative and poor oncological outcomes needs to be evaluated. The main aim of present study was to investigate the association of PM with perioperative clinical outcomes following EH. To do this, the effect of PM on intraoperative blood loss, morbidity, and posthepatectomy hemorrhage (PHH) was investigated. Additionally, the impact of PM on long-term outcomes was evaluated.

## Results

### Demographic and perioperative clinical data

**Figure 1:**
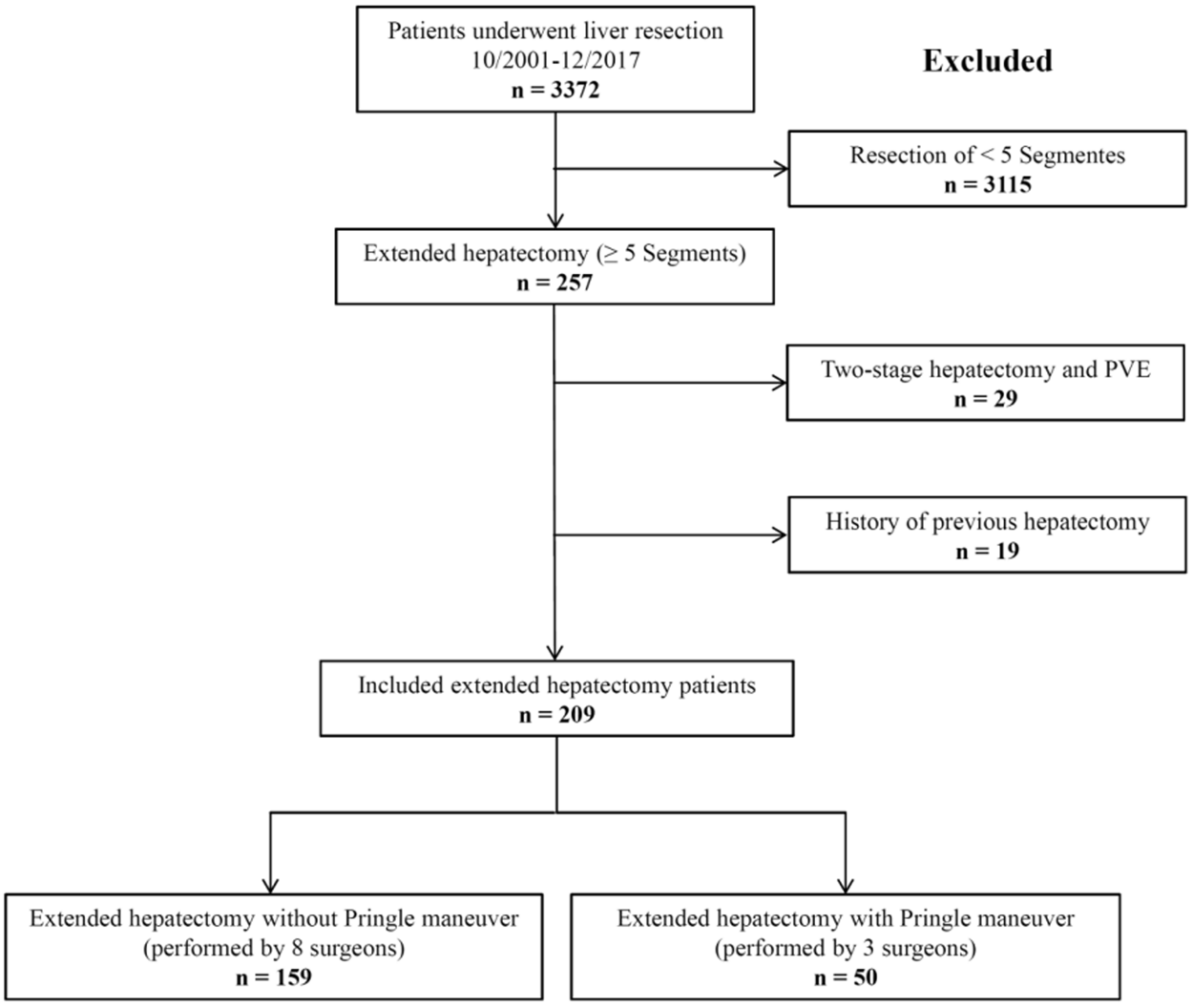
Flow diagram showing the study inclusion and exclusion criteria.

As shown in Fig. 1, 209 patients were included in this study. Baseline demographic and clinical data of patients is presented in Table 1. The mean age of patients was 60.0 ± 12.0 years and 51.2% were male. Primary hepatic malignancies were the most common indication for EH (n = 113, 54.1% of patients). Eight surgeons performed EH without the PM in 76.1% of patients and three surgeons performed EH with the PM in the remaining 23.9% of patients. There were no significant differences in demographic data, including age, sex, body mass index (BMI), and American Society of Anesthesiologists (ASA) class, between patients who underwent EH with or without the PM. However, 48.0% of patients (n = 24) who underwent EH without the PM were diagnosed with liver metastatic disease, while 22.0% of patients (n = 35) in the PM group were operated because of hepatic metastasis (*p* = 0.002). Therefore, more patients in the PM group received preoperative systemic chemotherapy compared with patients in the without PM group (60.0% vs. 33.3%, *p* = 0.001). Graphical presentation of the propensity score (PS) confirmed nearly complete overlap in the distribution of PS among the exposed and unexposed groups. Numerical diagnostics using Rubin’s criteria of absolute mean difference and variance ratios further validated the appropriateness of the estimated scores^19^. Intra- and postoperative data of included patients are presented in Tables 2 and 3, respectively.

**Table 1:** Demographic and preoperative clinical data of included patients.

Variables	Total (n = 209)	Without PM (n = 159)	With PM (n = 50)	*p*
Age (years)	60.0 ± 12.0	60.5 ± 12.3	58.4 ± 10.7	0.269
Sex Female/male	102/107	80/79	22/28	0.517
BMI (kg/m^2^)	25.4 ± 4.5	25.3 ± 4.4	26.0 ± 4.9	0.391
ASA class Class 1 Class 2 Class 3	7 (3.3%) 116 (55.5%) 86 (41.2%)	6 (3.7%) 85 (53.5%) 68 (42.8%)	1 (2.0%) 31 (62%) 18 (36%)	0.527
Cirrhosis	5 (2.4%)	3 (1.9%)	2 (4.0%)	0.595
Indication of hepatectomy Primary malignancy Cholangiocarcinoma Intrahepatic Klatskin type Hepatocellular carcinoma Colorectal liver metastasis Other liver diseases	113 (54.1%) 97 (46.4%) 56 (26.8%) 41 (19.6%) 16 (7.7%) 59 (28.2%) 37 (17.7%)	94 (59.1%) 83 (52.2%) 46 (28.9%) 37 (23.3%) 11 (6.9%) 35 (22.0%) 30 (18.9%)	19 (38.0%) 14 (28.0%) 10 (20.0%) 4 (8.0%) 5 (10.0%) 24 (48.0%) 7 (14.0%)	**0.002**
Preoperative chemotherapy Yes	83 (39.7%)	53 (33.3%)	30 (60.0%)	**0.001**

PM: Pringle maneuver; BMI: body mass index; ASA: American Society of Anesthesiologists.

All data were presented as mean (standard deviation) or n (%).

**Table 2:** Intraoperative data of included patients.

Variables	Total (n = 209)	Without PM (n = 159)	With PM (n = 50)	*p*
Transection technique Stapler LigaSure Clamp-crush CUSA	148 (70.8%) 24 (11.5%) 21 (10.0%) 16 (7.7%)	115 (72.3%) 19 (11.9%) 15 (9.4%) 10 (6.4%)	33 (66.0%) 5 (10.0%) 6 (12.0%) 6 (12.0%)	0.530
Side of resection Right Left	147 (70.3%) 62 (29.7%)	112 (70.4%) 47 (29.6%)	35 (70.0%) 15 (30.0%)	0.999
Intraoperative blood loss (L)	1.5 ± 1.4	1.7 ± 1.6	1.0 ± 0.7	**<0.001**
Excessive intraoperative bleeding (≥ 1,500 ml)	78 (37.3%)	69 (43.4%)	9 (18.0%)	**0.001**
Intraoperative RBC/FFP transfusion Patient Unit	65 (31.1%) 2.3 ± 5.2	56 (35.2%) 2.6 ± 5.6	9 (18.0%) 1.2 ± 3.4	**0.023** **0.035**
Operation time (hours)	4.9 ± 1.8	5.0 ± 1.9	4.6 ± 1.5	0.180

PM: Pringle maneuver; RBC: red blood cells; FFP: fresh-frozen plasma; CUSA: Cavitron Ultrasonic Surgical Aspirator.

All data were presented as mean (standard deviation) or n (%).

**Table 3:** Postoperative data of included patients.

Variables	Total (n = 209)	Without PM (n = 159)	With PM (n = 50)	*p*
Postoperative RBC transfusion Patient Unit	38 (18.2%) 0.7 ± 1.9	36 (22.6%) 0.8 ± 2.1	2 (4.0%) 0.1 ± 0.4	**0.002** <**0.001**
Postoperative FFP transfusion Patient Unit	26 (12.4%) 0.8 ± 2.4	25 (15.7%) 1.0 ± 2.7	1 (2.0%) 0.1 ± 0.6	**0.007** <**0.001**
ICU stay (days)	8.9 ± 14.7	9.9 ± 16.1	5.9 ± 8.3	**0.023**
Hospitalization (days)	24.3 ± 17.9	25.5 ± 18.9	20.1 ± 13.8	0.088
Posthepatectomy hemorrhage* Grade A Grade B Grade C	41 (19.6%) 26 (63.4%) 9 (22.0%) 6 (14.6%)	38 (23.9%) 23 (60.5%) 9 (23.7%) 6 (15.8%)	3 (6.0%) 3 (100%) 0 (0.0%) 0 (0.0%)	**0.004** 0.393
PHLF ^†^ Grade A Grade B Grade C	60 (28.8%) 16 (26.7%) 16 (26.7%) 28 (46.6%)	46 (28.9%) 11 (23.9%) 12 (26.1%) 23 (50.0%)	14 (28.6%) 5 (35.7%) 4 (28.6%) 5 (35.7%)	0.999 0.420
Major morbidity ^‡^	50 (23.9%)	46 (28.9%)	4 (8.0%)	**0.002**
Mortality	21 (10.0%)	17 (10.7%)	4 (8.0%)	0.403

ICU: intensive care unit; PM: Pringle maneuver; PHLF: posthepatectomy liver failure.

*Based on the ISGLS definition;^†^Based on the ISGLS definition; ^‡^Grade III and IV based on the Clavien-Dindo classification.

All data were presented as mean (standard deviation) or n (%).

### Outcome measures

#### Intraoperative data

Stapler hepatectomy was the most used parenchymal transection technique (n = 148, 70.8% of patients) and 70.3% of patients (n = 147) underwent right EH. The median duration of PM was 19 minutes, with a range between 13 and 49 minutes. The mean operation time was 4.9 ± 1.8 hours. The mean intraoperative blood loss was 1.5 ± 1.4 L and 37.3% of patients had excessive intraoperative bleeding (≥ 1,500 ml). 31.1% of patients (n = 65) received a red blood cells (RBC)/fresh-frozen plasma (FFP) transfusion with the mean amount of 2.3 ± 5.2 units RBC/FFP during the operation. As shown in Table 2, there were no significant differences in parenchymal transection technique and side of resection between the PM and without PM groups. The mean operation time was also not significantly different between the two groups. Performing the PM decreased the mean intraoperative blood loss by more than 40% (1.7 ± 1.6 L vs. 1.0 ± 0.7 L, *p* > 0.001). The rate and amount of intraoperative RBC/FFP transfusion was reduced by around 50% by the PM (rate: 35.2% vs. 18.0%, *p* = 0.023; amount: 2.6 ± 5.6 units vs. 1.2 ± 3.4 units, *p* = 0.035). The PM decreased the rate of excessive intraoperative bleeding from 43.4% to 18.0% (*p* = 0.001). Multivariate analysis revealed that the PM (OR = 0.27, 95% CI 0.10–0.70, *p* = 0.007) was an independent factor associated with excessive intraoperative bleeding (Table 4). The risk of excessive intraoperative bleeding was about fourfold higher in patients who underwent EH without the PM.

**Table 4:** Multivariate analysis of factors associated with excessive intraoperative bleeding (≥1,500 ml) after extended hepatectomy after propensity score adjustment.

Variables	OR	95% CI	*P*
Age ≤ 40 years	Reference	Reference	Reference
40–70 years	0.38	0.07–2.04	0.403
>70 years	0.05	0.01–0.37	**0.001**
Sex (male vs. female)	1.46	0.71–3.00	0.301
BMI < 25 (kg/m^2^)	Reference	Reference	Reference
25–30 (kg/m^2^)	1.31	0.52–3.31	0.904
≥30 (kg/m^2^)	1.94	0.70–5.38	0.323
ASA class (III vs. II and I)	0.97	0.49–1.92	0.926
Indication of hepatectomy (primary vs. others)	3.08	1.19–7.95	**0.020**
Preoperative platelet count <150 nL	18.67	3.18–109.74	**0.001**
Preoperative chemotherapy	2.52	0.79–7.96	0.117
Pringle maneuver	0.27	0.10–0.70	**0.007**
Transection technique (stapler vs. others)	0.41	0.18–0.92	**0.031**
Side of resection (right vs. left)	0.84	0.40–1.77	0.643

OR: odds ratio; CI: confidence interval; BMI: body mass index; ASA: American Society of Anesthesiologists; RBC: red blood cells; FFP: fresh-frozen plasma

#### Postoperative outcome

**Figure 2:**
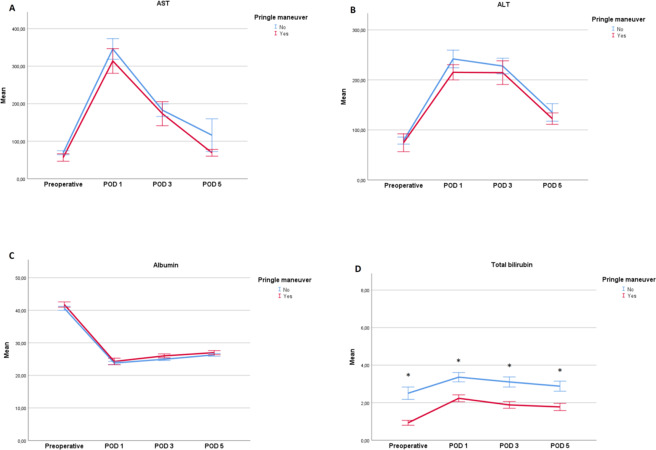
Changes in liver function before surgery and, 1, 3, and 5 days after surgery in patients who underwent extended hepatectomy with or without the Pringle maneuver. (**a**) aspartate aminotransferase (AST) (*p* for trend = 0.286), (**b**) alanine transaminase (ALT) (*p* for trend = 0.487), (**c**) albumin (*p* for trend = 0.221), and (**d**) total bilirubin (*p* for trend = 0.009). Error bars show standard error of the mean.

As shown in Fig. 2, results of liver function tests elevated early after EH and gradually decreased within 5 days. Repeated measures analysis of variance (ANOVA) revealed no significant differences in preoperative liver function and changes in liver function during the first 5 postoperative days between the two groups. Patients who underwent EH without the PM had significantly higher total bilirubin levels before and 1, 3, and 5 days after surgery compared with patients who underwent EH with the PM (Fig. 2b, *p* = 0.009). The mean duration of the intensive care unit (ICU) and hospital stay were 8.9 ± 14.7 days and 24.3 ± 17.9 days, respectively. The mean postoperative ICU stay in the PM group was 4 days shorter than in the without PM group (5.9 ± 8.3 days vs. 9.9 ± 16.1 days, *p* = 0.023). Statistically not significant but clinically important, the duration of hospitalization was also 5 days shorter in PM patients (Tables 3, 20.1 ± 13.8 days vs. 25.5 ± 18.9 days).

As shown in Tables 3, 23.9% of patients (n = 50) were faced with major morbidity (grade III and IV) after EH. The rate of major morbidity was significantly lower in patients with PM comparted to those without PM (28.9% to 8.0%, *p* = 0.002). No case of spleen rupture or portal vein embolism was reported in the PM group. Based on the multivariate analysis, the PM was significantly associated with reduced major morbidity after EH (Table 5, OR = 0.41, 95% CI 0.18–0.97, *p* = 0.041). PHH occurred in 28.8% of patients (n = 41) and most of them were classified as grade A PHH (n = 26, 63.4% of PHH). The PHH rate also decreased if the PM was used (23.9% vs. 6.0%, *p* = 0.004). Multivariate analysis revealed that the risk of PHH was significantly lower in patients who underwent EH with the PM (OR 0.22, 95% CI 0.06–0.79, *p* = 0.021) (Table 6). PHLF occurred in 28.8% of all patients (n = 60) and was not significantly different between the PM and without PM groups (Table 3). The mortality rate was 10.0% (n = 21) and was not significantly different between the PM and without PM groups (8.0% vs. 10.7%). Multivariate analysis after adjusting for PS revealed also no association between the PM and PHLF or mortality (data not shown).

**Table 5:** Multivariate analysis of factors associated with major morbidity after extended hepatectomy after propensity score adjustment.

Variables	OR	95% CI	*P*
Age ≤ 40 years	Reference	Reference	Reference
40–70 years	1.73	0.35–8.53	0.613
>70 years	5.29	1.01–27.72	**0.020**
Sex (male vs. female)	2.33	1.08–5.013	**0.030**
BMI < 25 (kg/m^2^)	Reference	Reference	Reference
25–30 (kg/m^2^)	0.81	0.28–2.32	0.887
≥30 (kg/m^2^)	0.76	0.28–2.08	0.755
Indication of hepatectomy (primary vs. others)	1.89	0.78–4.62	0.161
Preoperative platelet count <150 nL	12.36	2.53–60.43	**0.002**
Preoperative chemotherapy	0.60	0.20–1.83	0.371
Pringle maneuver	0.41	0.18–0.97	**0.041**
Transection technique (stapler vs. others)	0.92	0.42–2.01	0.830
Side of resection (right vs. left)	1.18	0.56–2.50	0.665

OR: odds ratio; CI: confidence interval; BMI: body mass index; ASA: American Society of Anesthesiologists; RBC: red blood cells; FFP: fresh-frozen plasma; AST: aspartate aminotransferase; ALT: alanine transaminase.

**Table 6:** Multivariate analysis of factors associated with posthepatectomy hemorrhage after extended hepatectomy after propensity score adjustment.

Variables	OR	95% CI	*p*
Age ≤ 40 years	Reference	Reference	Reference
40–70 years	1.43	0.29–7.03	0.280
>70 years	0.57	0.10–3.29	0.271
Sex (male vs. female)	0.63	0.29–1.40	0.257
BMI < 25 (kg/m^2^)	Reference	Reference	Reference
25–30 (kg/m^2^)	1.08	0.37–3.22	0.824
≥30(kg/m^2^)	0.90	0.25–3.22	0.827
ASA class (III vs. II and I)	1.23	0.58–2.60	0.599
Indication of hepatectomy (primary vs. others)	1.47	0.55–3.90	0.444
Preoperative platelet count <150 nL	1.51	0.41–5.60	0.539
Preoperative chemotherapy	1.58	0.47–5.37	0.464
Pringle maneuver	0.22	0.06–0.79	**0.021**
Transection technique (stapler vs. others)	1.59	0.62–4.06	0.333
Side of resection (right vs. left)	2.21	0.86–5.69	0.101

OR: odds ratio; CI: confidence interval; BMI: body mass index; ASA: American Society of Anesthesiologists; RBC: red blood cells; FFP: fresh-frozen plasma.

#### Oncological outcome

**Figure 3:**
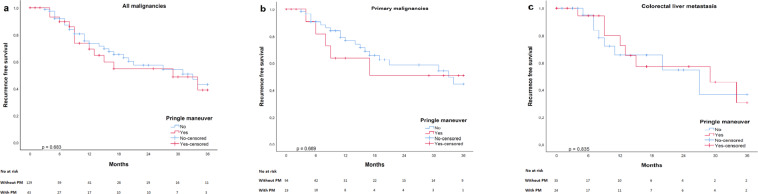
Kaplan-Meier survival curves illustrating 3-year recurrence-free survival in (**a**) all liver malignancies, (**b**) primary liver tumors and (**c**) colorectal liver metastasis. No significant difference in 3-year recurrence-free survival after extended hepatectomy was found between patients with and without the Pringle maneuver.

The median follow-up period for the entire cohort was 11 (range: 0–120) months. After excluding the patients with benign liver disease, the 3-year recurrence free survival was 41.8% in the total cohort. As shown in Fig. 3a, patients in the PM group had a 38.9% recurrence free survival and patients in the no PM group had a 43.0% recurrence free survival 3 years after EH (log-rank *p* = 0.683). Further sub-analysis revealed also no significant difference in recurrence free 3-year survival between PM and no PM groups in patients with primary liver malignancies (50.9% vs. 44.5%, and p = 0.669, Fig. 3b) and those with colorectal liver metastasis (30.5% vs. 36.5%, and p = 0.835, Fig. 3c).

## Discussion

Hepatic vascular occlusion methods, mostly the PM, are still frequently used by surgeons to control bleeding during liver resection and to decrease perioperative blood transfusion^20–23^. However, some studies have revealed that liver resection can be performed safely without using the PM^11,12^. Excessive intraoperative bleeding and vascular occlusion are both associated with an increased risk of postoperative morbidity and mortality. Therefore, best liver resection outcomes can be achieved when an operation is performed without hepatic vascular occlusion but with minimal blood loss and no blood transfusion. Today, with remarkable advances in surgical techniques and instruments, along with optimized anesthesia and intraoperative hemodynamic support, excellent outcomes have been achieved following liver resection without vascular clamping in high volume centers^11,12^. Nevertheless, the rates of laparoscopic and robotic major liver resections have increased, and controlling blood loss during these minimally invasive surgeries is difficult; to address this, several studies have recently been published to introduce different methods of the PM in laparoscopic or robotic surgeries^16–18^. In addition, the risk of intraoperative bleeding, perioperative blood infusion, postoperative complications, and mortality after EH are still considerable^5,24,25^.

Excessive intraoperative bleeding is inevitable in some patients who undergo EH. Therefore, it was hypothesized that reducing blood loss and preventing blood transfusion using the PM may outweigh the disadvantages of this method in patients who undergo EH. To test this hypothesis, the patient outcome after resection of ≥ five liver segments using the PM was investigated.

The results of the present study revealed that patients who underwent EH with the PM had significantly lower intraoperative bleeding and received less intraoperative RBC/FFP transfusion. Furthermore, the rate of excessive intraoperative bleeding was lower in the PM group. The hepatectomy was performed using staplers in about 70% of patients, and stapled hepatectomy was associated with a lower rate of excessive intraoperative bleeding. Similar to our findings, recent randomized-controlled trials have demonstrated that blood loss was lower during stapler hepatectomy compared with blood loss during other liver resection methods^26–28^. This indicates that the PM together with stapler hepatectomy may decrease intraoperative blood loss and prevent intraoperative blood transfusion. Additionally, patients in the PM group had significantly lower PHH, which reflects the reduced need for postoperative transfusion compared with patients in the without PM group. Perioperative blood transfusion has increased the length of hospital stay, worsened postoperative outcomes, and increased morbidity in liver resection patients^29,30^.

The PM can have negative effects, such as hepatic ischemia-reperfusion injury, spontaneous spleen rupture, and portal vein embolism^31^. In the present collective of EH patients, spleen rupture and portal vein embolism were not observed in patients after the PM. This indicates that the PM is a safe procedure, especially when it is performed quickly. The central venous pressure was always kept below 5 mmHg during the operation, which may have helped prevent intraoperative bleeding^32^. To assess the adverse clinical effects of ischemia-reperfusion injury, the rate of PHLF between the two groups was compared and no differences were observed. This shows that ischemia-reperfusion injury caused by a short PM does not lead to clinically significant liver damage and PHLF. Patients who were operated with the PM also had a significantly shorter ICU stay and lower rate of major morbidity compared with those who were operated without the PM. This can be explained by less intraoperative bleeding, blood transfusion, and PHH^29,30^. Although the PM did not significantly affect hospital stay and mortality, a longer ICU stay and higher rate of major morbidity are associated with higher costs and an increased need for intervention or reoperation^33^.

From an oncological point of view, there was no significant difference in 3-year recurrence rate between the two groups. These findings are in line with those of recent studies, which demonstrated that the PM does not affect recurrence after hepatectomy for both primary^34,35^ and secondary liver malignancies^36,37^. Some studies have shown that prolonged PM may be associated with recurrence after hepatocellular carcinoma^38^ and colorectal liver cancer metastasis^39^, but a fast PM does not increase the risk. The median duration of the PM in the present study was less than 20 minutes. Conversely, blood loss during hepatectomy and subsequent perioperative blood transfusion has been associated with poor overall and disease-free survival in hepatocellular carcinoma patients^35,40^. Therefore, not only does a shorter PM not increase the recurrence rate but it may even reduce it by preventing excessive blood loss and need for a blood transfusion.

Results of a European survey on the application of vascular control in liver surgery revealed that excessive blood loss, major hepatectomy, non-anatomical resections, and proximity to large vessels or bile ducts were common indications for vascular clamping during liver resection^41^. Deciding to perform the PM during hepatectomy should be based on an individual bleeding risk assessment and operation technique and difficulties. Indeed, because the liver is more vulnerable to bleeding than to ischemia^29–31,35^, the PM should be considered for procedures with a high risk of excessive intraoperative bleeding, such as EH. However, the PM should be performed as quickly as possible to prevent clinically significant liver damage due to ischemia-reperfusion injury. The liver can tolerate a continuous inflow occlusion of up to 120 minutes^42^. Therefore, clamping to prevent bleeding during EH is worthwhile, but should be done as quickly as possible.

The non-randomized design is a limitation of the present study because of possible selection bias. However, as mentioned above, the decision to perform the PM was based on the surgeons’ preference and was not influenced by patient-related factors. Additionally, to minimize potential bias and estimate the independent effect of the PM on posthepatectomy outcome, PS analysis was performed and factors that may affect the outcomes were controlled.

In conclusion, performing the PM is justified during EH because an EH has a high risk of excessive intraoperative bleeding. The PM decreases intraoperative blood loss and transfusion, reduces PHH and major morbidity, shortens the ICU stay, and does not affect long-term recurrence after EH. Of course, the duration of PM should be kept as short as possible. Randomized-controlled trials are necessary to draw robust conclusions regarding the use of the PM during EH.

## Patients and methods

### Study population and design

This is a non-randomized, single-centre, comparative study on patients underwent EH with or without the PM. A total of 3,372 consecutive patients who underwent liver resection between October 2001 and December 2017 were investigated. As shown in the study flow diagram (Fig. 1), patients who underwent a two-stage hepatectomy, portal vein embolization, or previous hepatectomy were excluded. In the end, 209 adult patients who underwent EH (resection of five or more hepatic segments based on the Brisbane 2000 classification) were included in the present study. EH was performed by eleven attending hepatobiliary surgeons, who had at least 3 years of experience in liver surgery. Three of these surgeons routinely performed the PM in all patients, while the remaining eight surgeons did not perform the PM during EH. All data were analyzed from a prospectively collected liver database. The study protocol was approved by the university’s independent ethics committee (S-754/2018). The requirement for informed consent was waived by the independent ethics committee of the University of Heidelberg due to the retrospective nature of this study. All procedures were performed according to the most recent revision of the Declaration of Helsinki.

### Operative techniques

Midline incision with a right inferolateral extension or Mercedes star incision was used. An intraoperative ultrasonography was routinely performed to determine the location and resectability of the lesions. After mobilization of the liver, the hepatoduodenal ligament was surrounded by a silicon tube to perform the PM. An intermittent PM was performed whenever the PM duration exceeded 15 minutes. In this case, the hepatic inflow was intermittently clamped with cycles of 10 minutes of occlusion and subsequently 5 minutes of reperfusion, which were repeated until the end of the hepatic transection. None of the patients underwent total hepatic vascular exclusion. Then, according to the location of the tumor, right or left EH was performed using a stapler (EndoGIA Universal; Covidien, Minneapolis, USA), LigaSure™ vessel sealing system (Medtronic, Dublin, Ireland), clamp-crushing technique, or Cavitron ultrasonic surgical aspirator (CUSA, Söring GmbH; Quickborn, Germany). These transection methods are described elsewhere^26–28^. Central venous pressure was monitored and maintained between 0 and 5 mmHg to minimize blood loss during the operation.

### Endpoints and patient evaluations

#### Endpoints

The Main endpoint of this study is to investigate the association of PM with excessive intraoperative bleeding, postoperative morbidity, and PHH. Accordingly, excessive intraoperative bleeding was defined as more than 1,500 ml blood loss during the operation. Postoperative complications were assessed and graded based on the Clavien-Dindo classification^43^. Minor morbidity was defined as Grade I and II morbidities, and major morbidity was defined as grade III and IV morbidities. Furthermore, PHH was diagnosed and graded in accordance with the definition of the International Study Group of Liver Surgery (ISGLS)^44^.

#### Preoperative evaluations

Patients’ demographic and clinical data including age, sex, BMI, ASA class, indication of hepatectomy, preoperative chemotherapy, and laboratory assessments were recorded. All patients underwent contrast-enhanced computed tomography or magnetic resonance imaging to assess the tumor resectability and extent of the resection.

#### Intraoperative evaluations

Intraoperative data, including use and duration of the PM, transection techniques, side of resection, intraoperative blood loss, amount of transfused RBC/FFP, and duration of operation were recorded.

#### Postoperative evaluations

The rate and amount of FFP and RBC transfusion during hospital stay were reported. After surgery, the duration of ICU and hospital stays were recorded. To evaluate posthepatectomy liver function, aspartate transaminase, alanine aminotransferase, albumin, alkaline phosphatase, and total bilirubin levels were measured before and after EH. PHLF was evaluated based on the ISGLS definition and grading^45^. Mortality was defined as all-cause death occurring within the first 30 days after EH. Disease recurrences diagnosed within the first 3 years after EH were also recorded.

### Statistical analysis

Continuous data were presented as means ± standard deviations or standard error of the means and categorical data were presented as frequencies and proportions. Continuous data were compared using Student’s t test and categorical data were analyzed using chi-square or Fisher’s exact test. A repeated measures ANOVA model was used to compare the overall differences among laboratory changes between the two groups. We used PS to account for confounding. PS was defined as the probability of being exposed (i.e. undergoing the PM) conditional on the relevant confounding variables. We considered age, sex, BMI, type of lesion, surgical indication, platelet count, chemotherapy, method and side of resection as potential confounding variables (Table 7). Logistic regression was used to estimate the conditional probabilities which then were classified into quintiles. The outcome logistic regression model included the PS quintiles, as well as all the individual confounders. Inclusion of individual confounders allows for the model to pick up residual confounding within each quintile. Further, this ‘doubly robust’ technique makes the results less susceptible to model misspecification in the PS or the outcome regression^46^. The cut-offs for age were chosen from the inflection points in the relationship between age and PM estimated from a generalized additive model to account for non-linearity. The cut-offs for BMI were defined as 25 kg/m^2^ and 30 kg/m^2^. Data preparation and cleaning was done in IBM SPSS Statistics for Windows version 22.0 (IBM Corp, Armonk, NY). All other statistical analyses were conducted using SAS version 9.4 (SAS Institute Inc., Cary, NC). A two-sided p value <0.05 was considered significant in all tests.

**Table 7:** Demographic, preoperative and intraoperative data of the patients included in propensity match analysis.

Variables	Without PM	With PM	Mean difference	*p*
	(n = 159)	(n = 50)		
Age (years)	60.5 ± 12.3	58.4 ± 10.7	2.151	0.269
Sex				0.517
Female/male	80/79	22/28	—	
BMI (kg/m^2^)	25.3 ± 4.4	26.0 ± 4.9	−0.755	0.391
Indication of hepatectomy			—	**0.002**
Primary malignancy	94 (59.1%)	19 (38.0%)		
▪ Cholangiocarcinoma	83 (52.2%)	14 (28.0%)		
✓ Intrahepatic	46 (28.9%)	10 (20.0%)		
✓ Klatskin type	37 (23.3%)	4 (8.0%)		
▪ Hepatocellular carcinoma	11 (6.9%)	5 (10.0%)		
Colorectal liver metastasis	35 (22.0%)	24 (48.0%)		
Other liver diseases	30 (18.9%)	7 (14.0%)		
Preoperative chemotherapy	53 (33.3%)	30 (60.0%)	—	**0.001**
Transection technique			—	0.530
Stapler	115 (72.3%)	33 (66.0%)		
LigaSure	19 (11.9%)	5 (10.0%)		
Clamp-crush	15 (9.4%)	6 (12.0%)		
CUSA	10 (6.4%)	6 (12.0%)		
Side of resection			—	0.999
Right	112 (70.4%)	35 (70.0%)		
Left	47 (29.6%)	15 (30.0%)		

PM: Pringle maneuver; BMI: body mass index; CUSA: Cavitron Ultrasonic Surgical Aspirator.

All data were presented as mean (standard deviation) or n (%).

## Data Availability

The datasets generated during and/or analysed during the current study are available from the corresponding author on reasonable request.
